# Recent Advances in Polyoxometalate‒Metal Nanocluster Hybrids

**DOI:** 10.1002/chem.202500877

**Published:** 2025-05-04

**Authors:** Kentaro Yonesato, Daiki Yanai, Kazuya Yamaguchi, Kosuke Suzuki

**Affiliations:** ^1^ Department of Applied Chemistry School of Engineering The University of Tokyo 7‐3‐1 Hongo, Bunkyo‐ku Tokyo 113–8656 Japan; ^2^ Department of Advanced Materials Science Graduate School of Frontier Sciences The University of Tokyo 5‐1‐5 Kashiwanoha Kashiwa, Chiba 277‐8561 Japan

**Keywords:** atomically precise nanoclusters, catalysis, hybrid materials, metal nanoclusters, polyoxometalates

## Abstract

Polyoxometalates (POMs) are structurally well‐defined anionic metal–oxo clusters with diverse structural forms and distinctive physicochemical properties, including acidity/basicity, redox activity, and optical behavior. Over the past two decades, POMs have garnered increasing attention in metal nanocluster synthesis owing to their ability to impart unique structural and functional characteristics to these compounds. Initially, research in this field primarily focused on hybrid materials composed of POMs and metal nanoclusters stabilized by organic ligands. However, more recently, studies have demonstrated that POMs can themselves act as effective stabilizing ligands for the synthesis of atomically precise metal nanoclusters. These POM‐stabilized metal nanoclusters exhibit synergistic and cooperative properties of metal nanocluster and POM components, enabling novel applications in fields such as catalysis, photochemistry, sensing, and electrochemistry. Notably, recent investigations into POM–metal nanocluster hybrids have demonstrated their potential as atomically precise counterparts to metal nanoparticle catalysts supported on bulk metal oxides, exhibiting the unique cooperative catalysis. This review provides a concise overview of POM‒metal nanocluster hybrids, with an emphasis on recent advancements in synthetic methodologies, properties, and applications. Additionally, solid‐state synthetic strategies leveraging the rigid structures and redox properties of POMs for the controlled formation of metal nanoclusters are discussed.

## Graphical abstract

POMs are gaining attention in metal nanocluster synthesis for their ability to impart unique structural and functional roles. This review highlights recent advances in POM–metal nanocluster hybrids, categorized as follows: (a) hybrids of POMs and metal nanoclusters stabilized by organic ligands, (b) metal nanoclusters stabilized by POMs as ligands, and (c) solid‐state synthesis of metal nanoclusters using POM‐defined scaffolds.

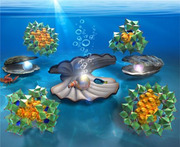

## 1 Introduction

**Figure 1:**
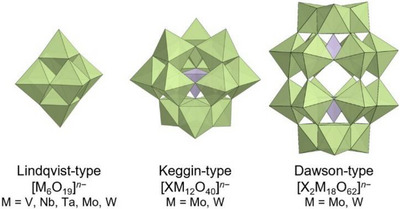
Anionic structures of Lindqvist‐type [M_6_O_19_]*^n^* ^−^ (M = V^5+^, Nb^5+^, Mo^6+^, Ta^5+^, and W^6+^), Keggin‐type [XM_12_O_40_]*^n^* ^−^, and Dawson‐type [X_2_M_18_O_62_]*^n^* ^−^ POMs (M = Mo^6+^ and W^6+^, X = main‐group elements from groups 13–16 or 3*d* transition metals). Color code: {MO_6_}, green octahedron; {XO_4_}, purple tetrahedron.

Polyoxometalates (POMs) are discrete anionic metal oxide clusters with diverse structural forms, including Lindqvist‐type [M_6_O_19_]*^n^* ^−^ (M = V^5+^, Nb^5+^, Mo^6+^, Ta^5+^, and W^6+^), Keggin‐type [XM_12_O_40_]*^n^* ^−^, and Dawson‐type [X_2_M_18_O_62_]*^n^* ^−^ structures (polyatom M = Mo^6+^, W^6+^; heteroatom X = main‐group elements from groups 13–16 or 3*d* transition metals; Figure 1).^[^ ^1^ ^]^ These clusters exhibit distinctive physicochemical properties—such as acidity/basicity, redox activity, catalytic activity, photochemical behavior, and electrochemical characteristics—depending on their structures, constituent elements, compositions, and electronic states. Furthermore, lacunary POMs, formed by the loss of specific {MO*_x_*} units (M = Mo, W) from plenary (stable) POM structures, function as versatile inorganic multidentate ligands. The basic oxygen atoms at the vacant sites of lacunary POMs enable coordination with a broad range of metal cations and metal oxide clusters. Accordingly, so far, various metal oxide clusters have been synthesized using lacunary POMs as templating ligands.^[^ ^2^, ^3^, ^4^ ^]^ These lacunary POMs have been frequently observed to exhibit cooperative and/or synergistic interactions with the incorporated metal oxide clusters. Moreover, studies have reported the chemical modification of POM frameworks through the substitution of their oxygen atoms with organic ligands,^[^ ^5^ ^]^ enabling their hybridization with a diverse array of organic molecules, including dyes (photosensitizers), biomolecules, and metal complexes.^[^ ^6^, ^7^, ^8^ ^]^ This versatility positions POMs as valuable molecular building units for the development of hybrid materials with tailored structures and properties.

Since the 1990s, the ability of POMs to stabilize nanoscale metal surfaces has garnered considerable research attention. For instance, Finke et al. elucidated the formation mechanism of Ir(0) nanoparticles using (TBA)_5_Na_3_[(1,5‐cyclooctadienyl)IrP_2_W_15_Nb_3_O_62_] (TBA = tetra‐*n*‐butylammonium) as a precursor, demonstrating that both the [P_2_W_15_Nb_3_O_62_]^9−^ POM and its countercation (TBA) function as stabilizing agents for Ir(0) nanoparticles.^[^ ^9^ ^]^ Since then, POMs have been widely used as stabilizing ligands for synthesizing various metal nanoparticles, including Au, Ag, Pt, Pd, Ir, Ru, and Rh.^[^ ^9^, ^10^ ^]^

Recent advancements in the liquid‐phase synthesis of nanoscale metal aggregates have facilitated the production of atomically precise metal nanoclusters (e.g., Au, Ag, Pt, and Pd), comprising 10 to a few hundred metal atoms and displaying uniform structural and electronic characteristics. This has been accomplished using organic ligands such as thiolates, phosphines, alkynyls, and *N*‐heterocyclic carbenes.^[^ ^11^ ^]^ These organic ligands stabilize metal nanoclusters either by directly coordinating to the metal surface or by forming monolayered metal–ligand assemblies, thereby facilitating their isolation, precise structural analysis, and novel applications. Despite unresolved doubts in the distinction between “nanoparticles” and “nanoclusters,”^[^ ^12^ ^]^ recent studies have revealed that small metal nanoclusters (typically < 2 nm in diameter) exhibit physicochemical properties that differ considerably from those of large nanoparticles (typically >5 nm in diameter). For instance, the metallic centers of small metal nanoclusters often exhibit partially oxidized states and carry intrinsic positive charges. Additionally, their atomic arrangements frequently deviate from those of conventional bulk‐metal structures, such as face‐centered cubic (fcc) and hexagonal close‐packed (hcp) configurations.^[^ ^12a^ ^]^ Most importantly, the unique properties and functions of metal nanoclusters—including catalytic activity, optical properties, and magnetism—vary widely depending not only on their size but also on their structures and electronic states.

The functionalization of metal nanoclusters with POMs offers several strategic advantages in both synthesis and applications: (1) The rigid and bulky POM surface, rich in oxygen atoms, can influence the spatial arrangement of metal atoms, promoting the formation of structurally unique nanoclusters. (2) The inherent negative charge of POMs electrostatically stabilizes the positively charged metal centers. (3) The electron‐donating character of POMs can fine‐tune the electronic states of metal nanoclusters, imparting novel electronic characteristics. (4) Synergistic interactions between POMs and metal nanoclusters can result in emergent physicochemical properties that are distinct from those of either POMs alone or metal nanoclusters stabilized by organic ligands. These enhanced properties expand their functional potential in catalysis, photochemistry, electrochemistry, magnetism, sensing, and biomedical applications.

**Figure 2:**
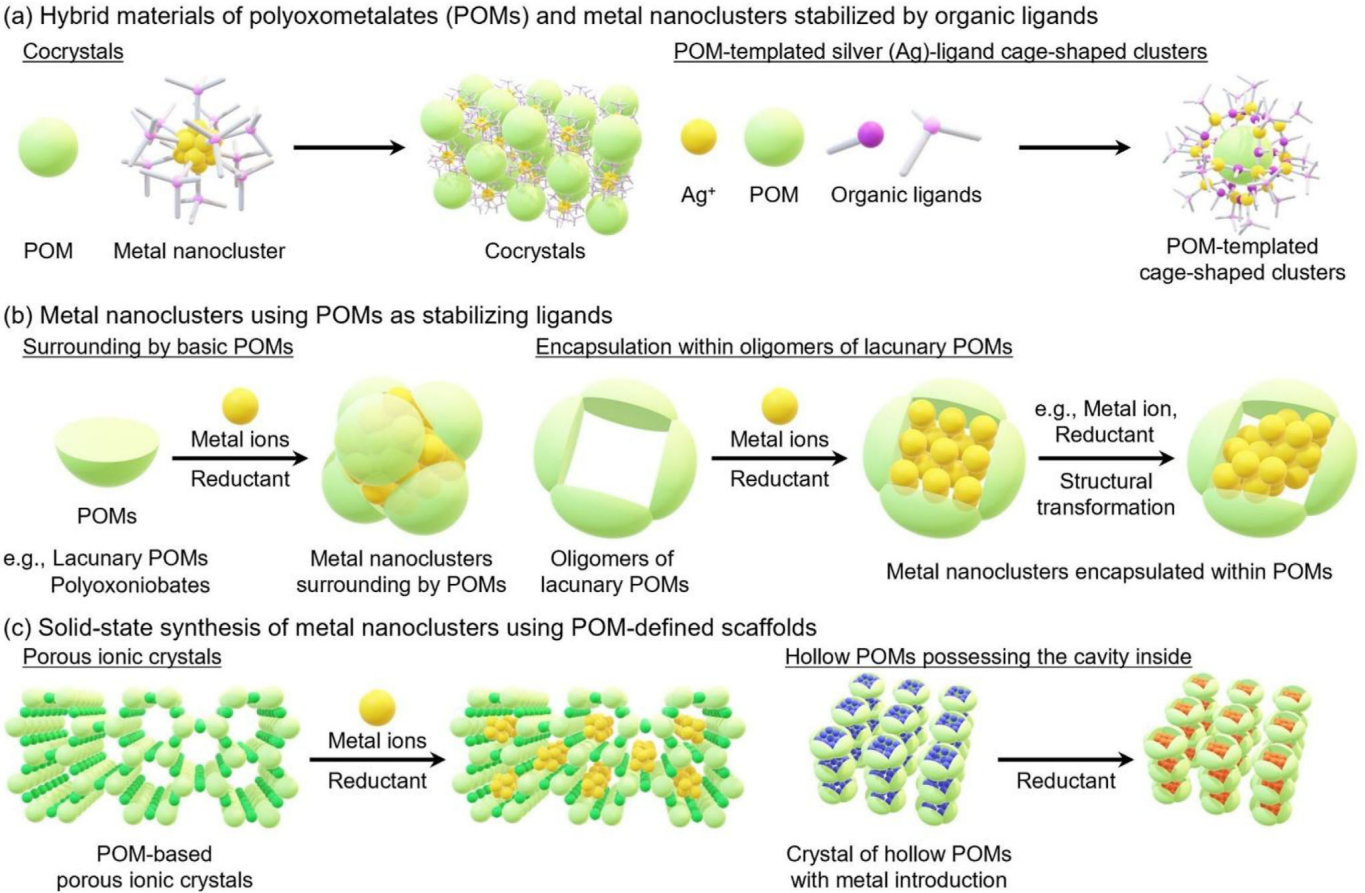
Focus of this review by section: (a) Hybrid materials composed of POMs and metal nanoclusters stabilized by organic ligands. (b) Metal nanoclusters using POMs as stabilizing ligands. (c) Solid‐state synthesis of metal nanoclusters using POM‐defined scaffolds.

This review, structured into five distinct sections, explores recent progress in the synthesis and applications of hybrid materials composed of atomically precise metal nanoclusters and POMs (Figure 2). Following the introductory section, the second section offers a concise overview of hybrid materials comprising POMs and metal nanoclusters stabilized by organic ligands, including cocrystals and POM‐templated Ag–organic ligand cage‐shaped clusters (Figure 2a). Research on these hybrid materials has demonstrated the structure‐directing potential of POMs, a property that has been extensively discussed in recent reviews.^[^ ^13^, ^14^ ^]^ The third section explores recent breakthroughs demonstrating that POMs with appropriate basicity—such as lacunary POMs and polyoxoniobates—can act as stabilizing ligands for atomically precise metal nanoclusters. These nanoclusters can be stabilized either by being sandwiched between POMs or encapsulated within the oligomeric structures of lacunary POMs (Figure 2b). Moreover, POM‐stabilized metal nanoclusters offer opportunities for further structural modifications, including self‐assembly, structural transformation, and metal substitution, facilitating the design of unique molecular hybrid structures. This review primarily focuses on recent advancements in the synthetic methodologies and properties of POM‐stabilized metal nanoclusters. Notably, these hybrid materials exhibit unique synergistic and/or cooperative properties that enable their application across various scientific fields. In particular, metal nanoclusters stabilized by POMs can be regarded as atomically precise analogs of supported metal nanoparticle catalysts. This parallel has led to investigations into their unique cooperative catalytic behavior, specifically the interactions between metal nanoclusters and the metal oxide frameworks of POMs. The fourth section of this review introduces solid‐state synthetic approaches for fabricating metal nanoclusters using POM‐defined scaffolds, such as porous ionic crystals and hollow POMs with nanosized cavities (Figure 2c). Finally, in the fifth section, the review concludes with a discussion on the future prospects and potential applications of atomically precise nanoclusters stabilized by POMs.

## 2 Hybrid Materials Comprising POMs and Metal Nanoclusters Stabilized by Organic Ligands

### 2.1 Cocrystals of POMs and Metal Nanoclusters

Among the various combinations of metal elements and organic ligands, Au‒phosphine nanoclusters represent one of the earliest and most extensively studied classes of monodisperse metal nanoclusters.^[^ ^15^ ^]^ These nanoclusters are typically cationic owing to the partial oxidation state of their Au core, which is stabilized by neutral phosphine ligands. This stabilization facilitates the formation of ionic cocrystals with POM anions.

In 2006, Jansen et al. reported the formation and crystal structure characterization of cocrystals composed of [Au_9_(PR_3_)_8_]^3+^ (R = Ph, *p*‐C_6_H_4_Me) and the Keggin‐type POM [PW_12_O_40_]^3−^, documenting the first example of a co‐crystalline hybrid consisting of metal nanoclusters and POMs.^[^ ^16^ ^]^ Since then, a wide range of Au, Ag, and alloy nanoclusters have been integrated with various POM anions, including Keggin‐type POMs (i.e., [XM_12_O_40_]*^n^* ^−^; X = Si, P, Co; M = Mo, W), Lindqvist‐type POMs (i.e., [M_6_O_19_]^2−^), and decavanadate [V_10_O_28_]^6−^, yielding structurally diverse co‐crystalline hybrids.^[^ ^13^, ^17^ ^]^

**Figure 3:**
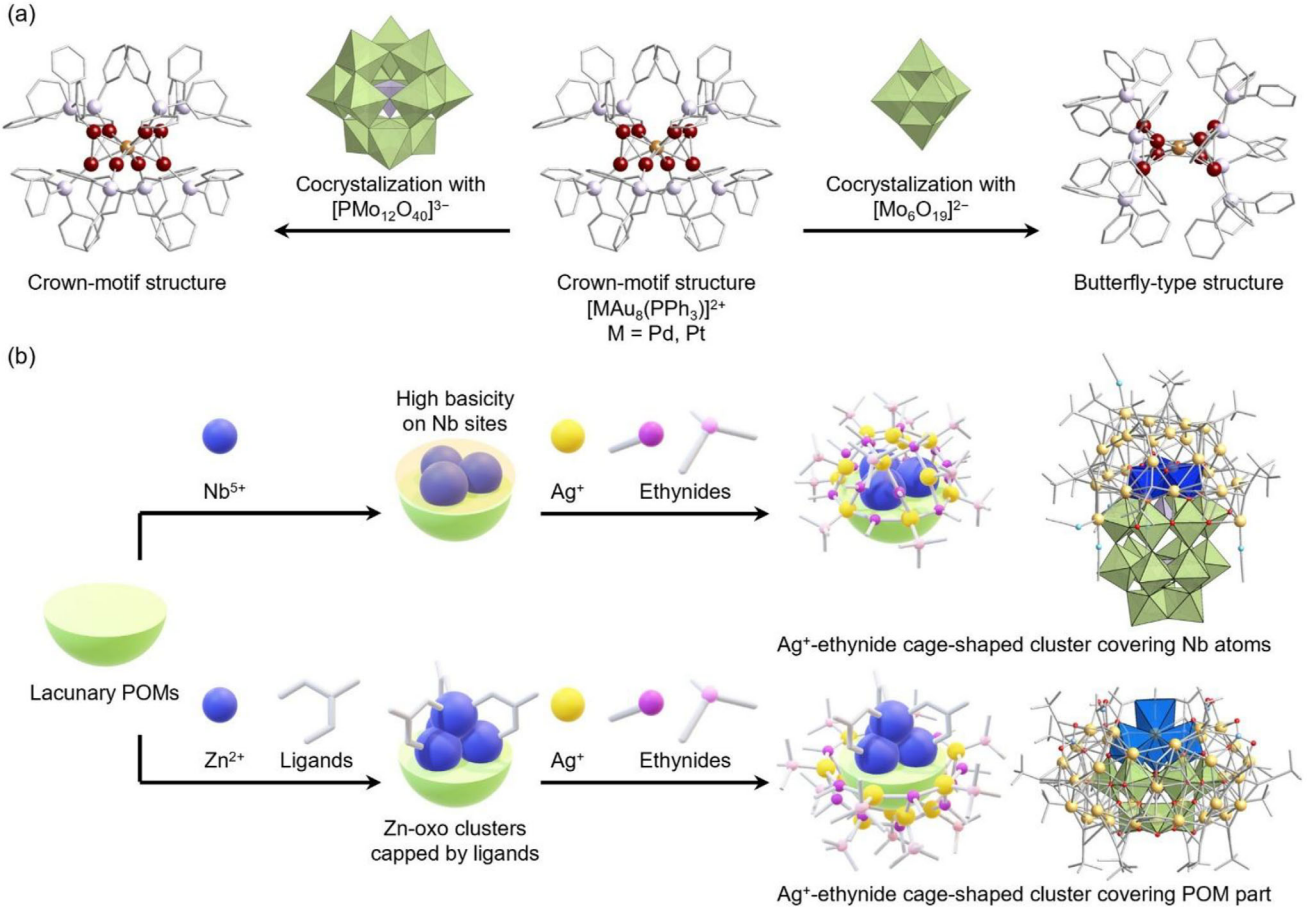
(a) Controlling [MAu_8_(PPh_3_)]^2+^ nanocluster isomerization by using different POMs as counteranions.^[^ ^18^ ^]^ (b) Modulating the structural morphology of POM‐templated Ag^+^‐ethynide clusters via the metal substitution of templating POMs.^[^ ^25^, ^26^ ^]^ The figures of the nanoclusters were made from the coordinates provided by the cited literatures.^[^ ^18^, ^25^, ^26^ ^]^ Color code: green, {MO_6_} (M = Mo, W); purple, {XO_4_}; dark blue, {NbO_6_}; light blue, {ZnO_6_}; dark red, Au; brown, Pd; light yellow, Ag; red, O; light blue, N; light purple, P.

Notably, despite the steric hindrance of phosphine ligands, which prevents direct coordination between metal nanoclusters and POMs, the hybridization of Au–phosphine nanoclusters and POMs presents a promising approach for tuning the spatial arrangement of metal atoms. Recently, Yamazoe et al. reported the isomerization of [MAu_8_(PPh_3_)_8_]^3+^ nanoclusters, wherein the structural transition from a crown‐like to a butterfly‐like configuration was induced through co‐crystallization with the Lindqvist‐type POM [Mo_6_O_19_]^2−^. This transformation was attributed to the stabilization of the metastable butterfly isomer within an NaCl‐type packing arrangement (Figure 3a).^[^ ^18^ ^]^ Conversely, when nitrate (NO_3_^−^) or the Keggin‐type POM [PMo_12_O_40_]^3−^ was used as a counteranion, the resulting cocrystals exhibited a CsCl‐type packing configuration, which did not promote isomerization. This finding indicates that the size and anionic charge of the counteranions play a pivotal role in dictating the structural behavior of these nanoclusters.

### 2.2 POM‐Templated Ag^+^‐Ligand Cage‐Shaped Clusters

Ag^+^‐organic ligand clusters can also be synthesized using anionic species as structural templates. Among the diverse range of anionic templates—including hydrides, halogens, chalcogens, and oxoanions^[^ ^19^, ^20^ ^]^—POMs offer distinct advantages owing to their high negative charge and the abundance of basic oxygen atoms (O^2−^) within their rigid, bulky frameworks. These characteristics allow POMs to influence the spatial arrangement of metal cations through direct coordination, imparting unique physicochemical properties to the resulting nanostructures.

Since the pioneering study of Mak et al. in 2009, which demonstrated the synthesis of the {Ag_40_(C ≡ C*^t^* Bu)_20_} cage‐type cluster templated by [V_10_O_28_]^6−^ and [Mo_6_O_22_]^8−^ anions,^[^ ^21^ ^]^ numerous analogous cage‐type clusters have been synthesized using various organic ligands—including alkynyl, thiol, phosphine, and carboxylate—in conjunction with POM templates.^[^ ^22^ ^]^ Several comprehensive reviews have also examined the progress in the synthesis, structural characteristics, and properties of these clusters.^[^ ^14^ ^]^

In this review, we provide a concise overview of synthetic approaches for controlling the morphology of Ag^+^–ethynide cage‐type clusters by leveraging POM design. Barring a few exceptions—such as the {Ag_42_(CO_3_)(C ≡ C*^t^* Bu)_27_(MeCN)} cage‐type cluster sandwiched between two [CoW_12_O_40_]^6−^ anions (Jansen et al., 2010)^[^ ^23^ ^]^ and the {Ag_43_(*^t^* BuC ≡ C)_29_(CN)(MeCN)(H_2_O)} cage‐type cluster partially encapsulating a [P_5_W_30_O_110_]^15−^ anion (Sun et al., 2021) — ^[^ ^24^ ^]^ Ag^+^‒ethynide cages typically form a complete outer layer around POM templates. Substituting metal centers within POMs provides an effective strategy for modulating the structural characteristics of Ag^+^–ethynide cage‐type clusters by altering the reactivity of oxygen atoms. In 2015, Ozeki et al. reported the synthesis of an {Ag_25_{C ≡ C*^t^* Bu)_3_}_16_(MeCN)_4_} cage‐type cluster using the niobium‐substituted POM [P_2_W_15_Nb_3_O_62_]^9−^ as an anionic template, wherein three tungsten atoms of the Dawson‐type POM [P_2_W_18_O_62_]^6−^ were replaced by niobium atoms.^[^ ^25^ ^]^ Given the increased basicity of the niobium‐oxide sites, Ag ions selectively coordinated to these regions, creating a hemispherical cage‐type structure (Figure 3b). In contrast, Li and Zheng et al. recently synthesized an Ag^+^–ethynide cage‐type cluster featuring an aperture by employing cubane‐like tetranuclear 3*d*‐metal‐incorporated POMs.^[^ ^26a^ ^]^ In these structures, the terminal oxygen atoms (M = O motifs) within the tetranuclear 3*d*‐metal oxide segments were entirely replaced by bridging acetate ligands, effectively preventing Ag ion coordination. Similarly, Lv and Yang reported the synthesis of Ag^+^‐ethynide cage‐type cluster using Co^2+^‐introduced POM [Co_4_(CH_3_COO)_2_(OH)_3_(H_2_O)SiW_9_O_33_]_2_ as a template, which was formed in‐situ by the reaction of trilacunary Keggin‐type POM [SiW_9_O_34_]^10−^ and Co^2+^ ions.^[^ ^22d^ ^]^

These reports highlight the versatility of POMs as templates, with their metal composition and charge playing key roles in directing the formation of Ag^+^–organic ligand cage‐type structures. Notably, POM‐templated Ag–organic ligand cage‐type clusters generally lack the defining characteristics of metal nanoclusters, such as metallic bonding character and super atomic electronic structures. This is because the Ag ions in these systems are typically restricted to a +1 oxidation state. To date, only two reports have documented POM‐templated Ag–organic ligand cage‐type compounds incorporating Ag^0^ atoms. For instance, in 2019, Zhuang, Sun, and co‐workers reported a rare example of an [M_7_O_26_]^10−^‐templated Ag‒organic ligand cage‐type cluster featuring central {Ag_10_} kernels sandwiched between two [M_7_O_26_]^10−^ anions (M = Mo, W). These {Ag_10_} kernels contained four free electrons, making the cluster one of the few known structures incorporating Ag^0^ atoms and metallic Ag─Ag bonds.^[^ ^27^ ^]^

## 3 Metal Nanoclusters Stabilized by POMs

### 3.1 Metal Nanoclusters Stabilized by Lacunary POMs

In both co‐crystallized complexes and anion‐templated Ag–organic ligand clusters, metal nanoclusters need to be stabilized by organic ligands to maintain structural integrity. However, as discussed in the introduction section, POMs themselves have the potential to function as stabilizing ligands for metal nanoclusters owing to their high negative charge and the abundance of oxygen atoms on their surfaces. While a wide variety of POMs that incorporate noble metal cations, such as Au, Ag, Pt, Pd, Ru, Rh, and Ir, has been reported,^[^ ^28^, ^29^ ^]^ this concept paper focuses on the metal nanoclusters that possess zerovalent metal atoms and metallic interactions.

The successful synthesis of Ag nanoclusters stabilized exclusively by POMs—without the involvement of organic ligands—was reported by Mizuno and some of the authors of this concept paper in 2013.^[^ ^30^ ^]^ They successfully obtained octahedral {Ag_6_}^4+^ nanoclusters sandwiched between two divacant lacunary silicotungstate [γ‐SiW_10_O_36_]^8−^ anions. However, synthesizing larger Ag nanoclusters using only POMs as stabilizing ligands has remained challenging, likely owing to the weak interactions between Ag atoms and the oxygen atoms of POMs. These weak interactions promote undesirable decomposition and aggregation of Ag atoms.

**Figure 4:**
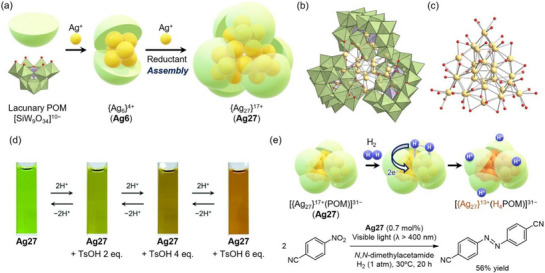
(a) Schematic representation of **Ag27** synthesis via self‐assembly. Crystal structures of **Ag27**: (b) anionic structure of [Ag_27_(Si_2_W_18_O_66_)_3_]^31−^) and (c) central structure of {Ag_27_}^17+^. The figure of the nanocluster was made from the coordinates provided by the cited literature.^[^ ^31^ ^]^ Color code: green, {WO_6_}; purple, {SiO_4_}; light yellow, Ag; red, O. (d) Color change of an **Ag27** acetonitrile solution upon the addition of *p*‐torylsulfonic acid (TsOH). Reprinted from the cited literature.^[^ ^32^ ^]^ Copyright 2022 Royal Chemical Society. (e) Schematic representation of the hydrogen dissociation and photocatalytic hydrogenation of nitroarenes using **Ag27**.^[^ ^33^ ^].^

In 2019, we reported the synthesis of an atomically precise, stable trefoil‐propeller‐shaped {Ag_27_}^17+^ nanocluster surrounded by three open‐Dawson‐type C‐shaped silicotungstate [Si_2_W_18_O_66_]^16−^ anions ([{Ag_27_}^17+^(Si_2_W_18_O_66_)_3_]^31−^; **Ag27**).^[^ ^31^ ^]^ Initially, a disilver‐containing C‐shaped anion (**Ag2**) formed via the dehydration condensation of two trivacant lacunary silicotungstate [α‐SiW_9_O_34_]^10−^ anions in the presence of Ag acetate (Figure 4a). Upon further addition of Ag^+^ ions in *N,N*‐dimethylformamide (DMF), acting as both a solvent and mild reductant, **Ag27** was successfully obtained through the self‐assembly of {Ag_6_}^4+^ species captured by the C‐shaped POM (**Ag6**). Notably, the {Ag_27_}^17+^ nanocluster comprises a central {Ag_9_} core arranged in an hcp structure, three octahedral {Ag_6_} units, and three Ag atoms bridging the octahedral {Ag_6_} moieties (Figure 4b,c). A density functional theory (DFT) study revealed that the 10 valence electrons of the {Ag_27_}^17+^ nanocluster occupy *p*‐ or *d*‐orbital‐like molecular orbitals that are delocalized over the entire {Ag_27_}^17+^ nanocluster, demonstrating the superatomic character of **Ag27**. In the ultraviolet‒visible (UV‒Vis) spectrum of **Ag27** in acetonitrile, several characteristic absorption bands were apparent in the visible region. A time‐dependent DFT study attributed these absorption bands to both intra‐{Ag_27_} electron excitation (λ ≈ 600 nm) and charge transfer from the {Ag_27_} nanoclusters to the W 5*d* orbitals of the POM framework (λ ≈ 530 nm).

Owing to the hybrid structure of **Ag27**, which combines a nanocluster with polyoxotungstates, it exhibits several distinctive properties. For instance, it demonstrates exceptionally high stability in both solid and solution states, whereas conventional Ag nanoclusters are prone to decomposition and aggregation. Additionally, the electronic states of the {Ag_27_}^17+^ center can be modulated by the protonation state of the C‐shaped POMs. The addition of an acid (i.e., *p*‐toluenesulfonic acid; TsOH) inhibits electron donation from the C‐shaped POMs to the Ag center, thereby weakening electrostatic repulsion, redistributing the charge of {Ag_27_}^17+^, and inducing pronounced yet reversible changes in light absorption properties (Figure 4d).^[^ ^32^ ^]^

Metal nanoparticle or nanocluster catalysts supported on metal oxides frequently exhibit synergistic and cooperative reactivity at the interface, leading to enhanced catalytic activity and selectivity. Given that POM‐stabilized Ag nanoclusters can be regarded as atomically precise counterparts to metal nanoparticle catalysts supported on bulk metal oxides, they serve as a valuable platform for investigating unique cooperative reactivities and catalytic properties between Ag nanoclusters and the metal oxide frameworks of POMs.

Notably, **Ag27** exhibits unique reactivity, enabling the cleavage of dihydrogen molecules (H_2_) into protons and electrons (Figure 4e).^[^ ^33^ ^]^ Typically, H_2_ dissociation either follows a homolytic pathway (H_2_ → 2H atoms) on a metal surface or a heterolytic pathway (H_2_ → H^+^ + H^−^) at the interface between metal nanoparticles and metal oxide supports.^[^ ^34^ ^]^ Using electrospray ionization mass spectrometry, nuclear magnetic resonance (NMR) spectroscopy, elemental analysis, titration tests, and Ag K‐edge X‐ray absorption fine structure (XAFS) analysis, researchers observed that two H_2_ molecules react with **Ag27**, undergoing cleavage into four protons and four electrons. Among these, the protons are stored within the POM framework, while the electrons are retained within the {Ag_27_}^17+^ core, as represented by the reaction [(Ag_27_)^17+^(Si_6_W_54_O_198_)]^31−^ + 2H_2_ → [(Ag_27_)^13+^(H_4_Si_6_W_54_O_198_)]^31−^ (Figure 4e). Additionally, **Ag27** exhibits photocatalytic activity, facilitating visible‐light‐induced electron transfer from the {Ag_27_} nanocluster to the [Si_2_W_18_O_66_]^16−^ anions during the reduction of 4‐nitrobenzonitrilie to the corresponding azobenzene. Recently, Lv, Yang, and co‐workers reported a photocatalytic CO_2_ reduction system employing two trefoil‐propeller‐shaped Ag nanoclusters stabilized by [Si_2_W_18_O_66_]^16−^ anions: [Ag_27_(Si_2_W_18_O_66_)_3_], which is structurally identical to **Ag27**, and [Ag_24_(Si_2_W_18_O_66_)_3_], where the {Ag_24_} structure lacks the three bridging Ag atoms that interconnect the octahedral {Ag_6_} units in {Ag_27_} nanoclusters.^[^ ^35^ ^]^ Using Ru(bpy)_3_Cl_2_ as a photosensitizer, both the {Ag_24_} and {Ag_27_} nanoclusters exhibited catalytic activity during the reduction of CO_2_ to formic acid with high selectivity.

### 3.2 Metal Nanoclusters Encapsulated within Ring‐Shaped POMs

Despite the promising potential of POM‐stabilized Ag nanoclusters (e.g., **Ag27**), the steric hindrance of the rigid and bulky surface of the Ag center limits their catalytic utility, as the active sites on the nanocluster surface remain inaccessible. Notably, this challenge is not unique to POM‐stabilized nanoclusters but represents a general obstacle in the development of atomically precise metal nanocluster catalysts, including those stabilized by organic ligands, where creating an exposed metal surface remains difficult. The exposed metal surfaces of nanoclusters are expected to exhibit high reactivity owing to the presence of metal atoms in unsaturated coordination states. However, this same characteristic compromises their structural stability, often leading to undesirable growth and/or decomposition.

**Figure 5:**
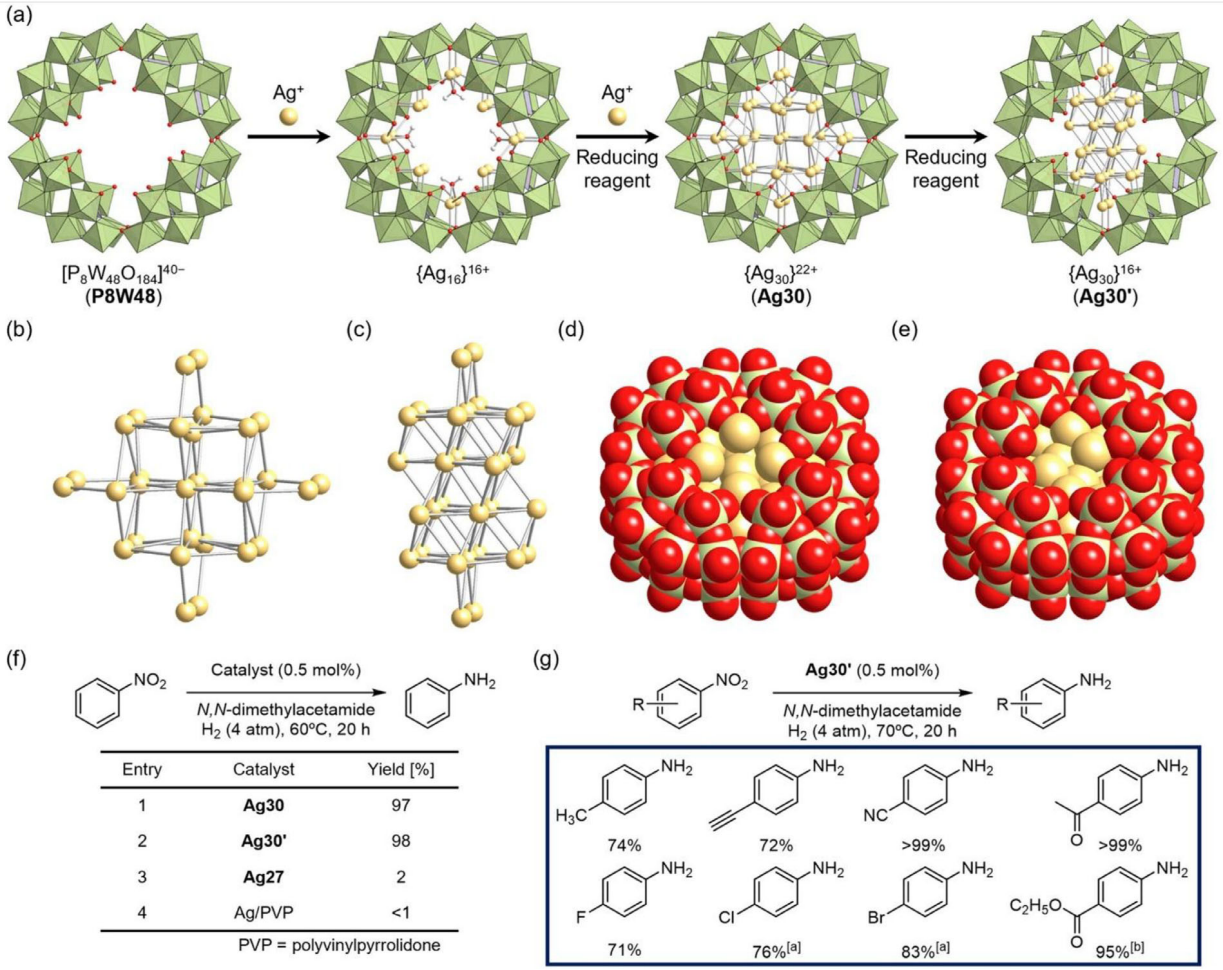
(a) Schematic representation of Ag_30_ nanocluster synthesis using the ring‐shaped POM **P8W48** as a stabilizing ligand. (b,c) Central {Ag_30_} structures of (b) bcc‐type **Ag30** and (c) fcc‐type **Ag30′**. (d,e) Space filling representations of (d) **Ag30** and (e) **Ag30′**. The figures of nanoclusters were made from the coordinates provided by the cited literature.^[^ ^36^ ^]^ Color code: green, {WO_6_}; purple, {PO_4_}; light yellow, Ag; red, O. (f) Effect of catalysts for nitrobenzene reduction to aniline, with **Ag30**, **Ag30′**, and **Ag27** nanoclusters (completely covered {Ag_27_} nanoclusters^[^ ^31^ ^]^) and PVP‐coated silver nanoparticles (Ag/PVP). (g) Substrate scope for **Ag30′**‐catalyzed selective nitroarene reduction. Reaction conditions: nitroarene (0.1 mmol), **Ag30′** (0.5 mol%), DMA (1 mL), H_2_ (4 atm), 70 °C and 15 h. ^[a]^H_2_ (8 atm), 60 °C and 20 hours. ^[b]^H_2_ (8 atm) and 5 hours. Reproduced from the cited literature.^[^ ^36^ ^]^ Copyright 2023 Nature Publishing Group.

Recently, we synthesized surface‐exposed Ag nanoclusters encapsulated within ring‐shaped POMs, which effectively protect the nanoclusters from undesirable aggregation and decomposition.^[^ ^36^ ^]^ Specifically, the ring‐shaped POM [P_8_W_48_O_184_]^40−^ (**P8W48**),^[^ ^37^ ^]^ which features a spacious cavity (∼1 nm in diameter) surrounded by abundant oxygen atoms, has previously been employed as a molecular template for incorporating various metal oxide clusters.^[^ ^38^ ^]^ Initially, we synthesized **P8W48** (**Ag16**), a complex containing 16 Ag^+^ ions, by reacting the TBA salt of **P8W48** with Ag acetate (Figure 5a). A subsequent reaction of **Ag16** with additional Ag acetate in DMF facilitated the formation of a distorted body‐centered cubic (bcc) {Ag_30_}^22+^ nanocluster ([{Ag_30_}^22+^(P_8_W_48_O_184_)]^18−^; **Ag30**; Figure 5b), containing an additional 14 Ag atoms relative to **Ag16**. Upon treating **Ag30** with tetra‐*n*‐butylammonium borohydride (TBABH_4_) as a reducing agent, the inner {Ag_30_}^22+^ nanocluster underwent a six‐electron reduction process, inducing structural rearrangement into the {Ag_30_}^16+^ nanocluster within **P8W48** ([{Ag_30_}^16+^(P_8_W_48_O_184_)]^24−^; **Ag30′**). In this reduced form, 26 Ag atoms adopted an fcc structure (Figure 5c). Notably, this reduction‐triggered structural isomerization from **Ag30** to **Ag30′** also proceeded in the solid state under an H_2_ gas atmosphere. In total, the number of valence electrons in **Ag30** and **Ag30′** was eight and 14, respectively, with delocalized electronic states spanning the entire nanocluster. DFT calculations of **Ag30** revealed that its superatomic orbitals exhibit degeneracies owing to its high symmetry (*D*_4_ _h_). For instance, in **Ag30**, molecular orbitals from the highest occupied molecular orbital (HOMO) to HOMO−2 and from the lowest unoccupied molecular orbital (LUMO) to LUMO+4 correspond to the superatomic 1*P* and 1*D* orbitals, respectively. In particular, a pair of HOMO−2 and HOMO−1 orbitals (equivalent to the *p*_x_ and *p*_y_ orbitals) and another pair of LUMO+3 and LUMO+4 (equivalent to *d*_yz_ and *d*_zx_ orbitals) are degenerate. In contrast, **Ag30′**, which exhibits lower symmetry (*C*_2_ _h_), displayed a more complex splitting of superatomic molecular orbitals. In this structure, the HOMO−2 to HOMO and LUMO to LUMO+1 were attributed to the superatomic 1*D* orbitals and W 5*d* orbitals, respectively. We recently discovered another {Ag_30_} nanocluster with an fcc‐type structure similar to **Ag30′** but with a different valence electron count (12‐electron system; {Ag_30_}^18+^) via a one‐step reaction involving **Ag16**, Ag acetate, and TBABH_4_.^[^ ^39^ ^]^

X‐ray crystallographic analysis confirmed that in these {Ag_30_} nanoclusters, an exposed Ag surface exists within the aperture of **P8W48** (Figure 5d,e).^[^ ^36^ ^]^ Typically, exposed metal surfaces in nanoclusters are highly susceptible to decomposition and/or agglomeration. However, **Ag30′** exhibits remarkable stability in solutions, likely owing to the protective effect of the rigid and bulky **P8W48** framework, which prevents undesirable structural transformations. This stabilization, combined with the exposed Ag surface and the interface between the Ag nanocluster and the metal oxide framework (**P8W48**), endows **Ag30′** with exceptional catalytic activity and selectivity in the hydrogenation of nitroarenes to their corresponding anilines. Given that neither **Ag27** nor Ag/polyvinylpyrrolidone exhibited catalytic activity under identical conditions (Figure 5f), the unique catalytic behavior of **Ag30′** likely originates from (1) the exposed Ag surface and/or the nanocluster–**P8W48** interface and (2) the distinct H_2_ cleavage properties of **Ag30′**, wherein H_2_ dissociates into protons and electrons (H_2_ → 2H^+^ + 2e^−^). In addition, supported Ag nanoparticle catalysts on metal oxides typically required a high H_2_ pressure and/or high temperature (Table 1, e.g., >10 atm H_2_ pressure, >100 °C), while **Ag30′** exhibited efficient catalytic activity under mild conditions (Figure 5f; H_2_ 4 atm, 60 °C).^[^ ^36^ ^]^ In addition, this catalytic system could be further applicable to the selective reduction of various nitroarenes (Figure 4e). When 4‐ethynylnitrobenzene was subjected to hydrogenation using conventional Ag nanoparticle catalysts, both the ethynyl (C ≡ C) and nitro (‐NO_2_) functional groups underwent hydrogenation via the homolytic (H_2_ → 2H atoms) and heterolytic (H_2_ → H^+^ + H^−^) cleavage of H_2_. Conversely, when **Ag30′** was used as a catalyst, selective nitro group hydrogenation was observed, while the ethynyl group remained unchanged (Figure 5g). Importantly, in situ XAFS analysis confirmed that **Ag30′** retains its structure under catalytic conditions and after the reaction.

**Table 1:** Comparison of the reaction conditions and catalytic activity in nitroarene reductions using Ag‐catalysts and H_2_ as the reductant.

Catalyst	Substrate	Pressure of H_2_ [atm]	Temperature [°C]	Yield [%]	Ref
**Ag30**	Nitrobenzene	4	60	97	[36]
**Ag30′**	Nitrobenzene	4	60	98	[36]
Ag/PVP^[^ ^a^ ^]^	Nitrobenzene	4	60	<1	[36]
Ag/SiO_2_	2‐Chloronitrobenzene	5	140	20	[40a]
Ag/SiO_2_	2‐Chloronitrobenzene	20	140	100	[40a]
Ag@C/ZrPP‐500^[^ ^b^ ^]^	Nitrobenzene	40	130	48	[40b]
Ag@C/ZrPP‐500^[^ ^b^ ^]^	Nitrobenzene	40	160	100	[40b]
Ag/Al_2_O_3_	4‐Nitrostyrene	30	160	96	[40c]
Ag@CeO_2_	4‐Nitrostyrene	6	110	98	[40d]
Ag/Al_2_O_3_	4‐Chloronitrobenzene	17	100	15	[40e]

^[a]^ PVP = polyvinylpyrrolidone.

^[b]^ ZrPP = zirconium pyrophosphate.

A key challenge in the development of atomically precise POM‐stabilized metal nanoclusters is extending the synthetic approach beyond Ag to other metals. For instance, Au nanoclusters have been extensively studied owing to their intrinsic stability and are typically stabilized by organic ligands such as thiolates, phosphines, and alkynyls.^[^ ^41^ ^]^ However, POM‐stabilized Au nanoclusters are yet to be realized, likely because of the weak interaction between gold cations (Au^+^, Au^3+^) and the oxygen atoms of POMs, making their incorporation into POM frameworks difficult. Although Kortz et al. have reported several polyoxoaurates,^[^ ^28^, ^42^ ^]^ Au‐substituted POMs—where metal sites (typically Mo^6+^, W^6+^) are replaced by Au atoms—have not yet been reported, despite their significant potential as precursors for POM‐stabilized Au nanoclusters.

**Figure 6:**
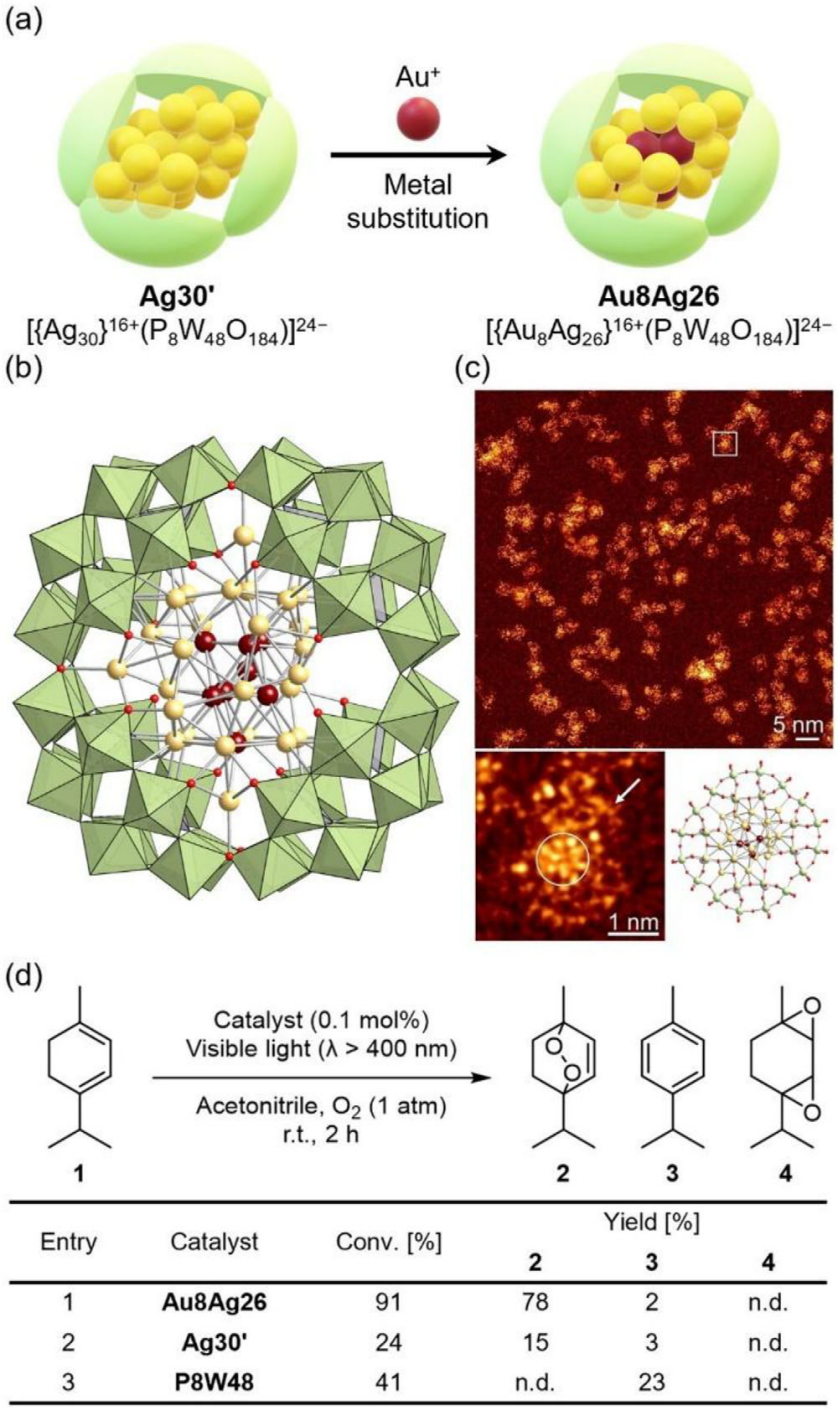
(a) Schematic of the synthesis of **Au8Ag26** via metal substitution between Au^+^ ions and **Ag30′**. (b) Anionic structure of **Au8Ag26**. The figure was made from the coordinates provided by the cited literature.^[^ ^43^ ^]^ Color code: green, {WO_6_}; purple, {PO_4_}; dark red, Au; light yellow, Ag; red, O. (c) Annular dark‐field scanning transmission electron microscopy (ADF‐STEM) image of **Au8Ag26**. Reprinted from the cited reference.^[^ ^43^ ^]^ Copyright 2024 John Wiley & Sons. (d) Photocatalytic oxidation of α‐terpinene via singlet oxygen (^1^O_2_) generation using **Ag8Ag26**, **Ag30′**, and **P8W48** as photocatalysts.

While the direct stabilization of single‐metal nanoclusters beyond Ag remains challenging, alloying provides a promising strategy for expanding the structural and functional diversity of POM‐stabilized nanoclusters. We recently reported the synthesis of a core‐shell‐type Au–Ag alloy nanocluster encapsulated within **P8W48** ([{Au_8_Ag_26_}^16+^(P_8_W_48_O_184_)]^24−^; **Au8Ag26**), wherein the electron‐rich central Ag^0^ sites were replaced by Au atoms through the reaction of **Ag30′** with a Au complex (Figure 6a). X‐ray crystallographic analysis and scanning transmission electron microscopy revealed that Au atoms were incorporated into **P8W48** by substituting the central Ag atoms of **Ag30′**, leading to the formation of a core‐shell Au–Ag alloy nanocluster (Figures 6b,c).^[^ ^43^ ^]^ To date, Au and Au–Ag alloy nanoclusters have been revealed to act as photosensitizers capable of generating singlet oxygen (^1^O_2_) under visible or near‐infrared light irradiations, thereby enabling selective oxidative transformations, such as the conversion of sulfides to sulfoxides and amines to imines.^[^ ^44^ ^]^ The Au–Ag alloy nanocluster within the ring‐shaped POM (i.e., **Au8Ag26**) exhibited enhanced activity and stability during the visible‐light‐induced photocatalytic oxidation of α‐terpinene via ^1^O_2_ generation, whereas **Ag30′** alone displayed lower reactivity and underwent decomposition under identical conditions (Figure 6d).

### 3.3 Metal Nanoclusters Stabilized by Other POMs

**Figure 7:**
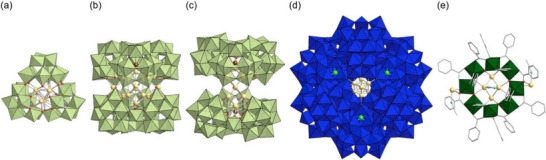
Anionic structures of Ag nanoclusters stabilized by POMs or titanium‐oxo clusters: (a) {Ag_7_} nanocluster encapsulated within a triangular anion [Si_3_W_27_O_96_]^18−^,^[^ ^45^ ^]^ (b) {Ag_14_} nanocluster sandwiched between two bowl‐shaped [Sb_3_ W_30_O_103_]^17−^,^[^ ^46^ ^]^ (c) {Ag_14_} nanocluster sandwiched between two bowl‐shaped [Sb_3_ W_30_O_102_]^15−^,^[^ ^46^ ^]^ (d) {Ag_8_} nanocluster stabilized by polyoxoniobate [Li_3_Nb_81_O_225_]^42−^,^[^ ^47^ ^]^ and (e) {Ag_6_} nanocluster encapsulated within the titanium‐oxo cluster [Ti_16_O_22_(PhCO_2_)]^6−^.^[^ ^48a^ ^]^ The figures of nanoclusters were made from the coordinates provided by the cited literatures.^[^ ^45^, ^46^, ^47^, ^48^ ^]^ Color code: green, {WO_6_}; purple, {SiO_4_}; dark blue, {NbO_6_}; dark green, {TiO_6_}; light yellow, Ag; brown, Sb; red, O; light green, Li; light blue, N.

Similar to **P8W48**, lacunary POMs can assemble into oligomeric structures with well‐defined internal cavities that serve as molecular templates, dictating the size and spatial arrangement of encapsulated metal nanoclusters. In an unexpected discovery, we found that adding excess Ag^+^ during the synthesis of **Ag2** ^[^ ^31^ ^]^ —comprising a C‐shaped POM [Si_2_W_18_O_66_]^16−^ dimer derived from the trivacant lacunary Keggin‐type POM [A‐α‐SiW_9_O_34_]^10^—led to the formation of a novel ring‐shaped triangular anion [Si_3_W_27_O_96_]^18−^ encapsulating an {Ag_7_}^5+^ nanocluster (Figure 7a).^[^ ^45^ ^]^ Three [A‐α‐SiW_9_O_34_]^10−^ units assembled to form this triangular POM, with one {SiW_9_} unit bridging the C‐shaped POM dimer via four shared oxygen atoms at their vacant sites. More recently, Lv, Yang, and co‐workers reported the synthesis of two {Ag_14_} nanoclusters sandwiched between two bowl‐shaped [Sb_3_ W_30_O_102_]^9−^ or [Sb_3_ W_30_O_103_]^11−^ anions,^[^ ^46^ ^]^ where the {Sb_3_ W_30_} anions formed through the bonding of one [B‐α‐SbW_9_O_34_]^9−^ and two [B‐β‐SbW_9_O_34_]^9−^ units via three {WO*_x_*} octahedra in distinct bonding modes (Figure 7b,c).

These examples demonstrate that lacunary polyoxotungstates (or their oligomeric derivatives) serve as effective stabilizing ligands owing to the basic oxygen atoms at their vacant sites and their inherent stability. Expanding the range of POM ligands could lead to the development of metal nanoclusters with novel synergistic properties and applications. Notably, Li, Zheng, and co‐workers recently reported the synthesis of a rhombic {Ag_8_} nanocluster sandwiched between two polyoxoniobate [Nb_81_O_225_]^45−^ anions, documenting the first instance of a basic niobium‐oxide framework acting as a stabilizing ligand for atomically precise Ag nanoclusters (Figure 7d).^[^ ^47^ ^]^ Similarly, Zhang et al. reported that titanium‒oxo nanoring clusters, in which titanium atoms are bridged by oxygen atoms and carboxylates, can encapsulate small Ag nanoclusters^[^ ^48^ ^]^ as well as a Cu nanocluster (Figure 7e).^[^ ^49^ ^]^

### 3.4 Stabilities of Metal Nanoclusters Stabilized by POMs

The stability of metal nanoclusters under various conditions is a crucial issue that must be carefully assessed when developing their properties and applications, as these characteristics are highly dependent on the structures and electronic states of metal nanoclusters. To date, the metal nanoclusters—particularly those composed of Ag and Au–Ag alloys—stabilized by POMs have frequently demonstrated high stability. This is one of the most fascinating features of POM‐stabilized metal nanoclusters and has contributed significantly to the development of their diverse properties, including optical properties, redox reactivity, and catalytic properties.

UV‐vis spectroscopy is a widely used and facile method for evaluating the stability of metal nanoclusters, as their spectra typically exhibitcharacteristic absorption bands that reflect their structural and electronic states. For example, while UV‐vis spectra of thiol‐stabilized Ag nanoclusters have been reported to significantly degradeduring a week in solution,^[^ ^50^ ^]^ those of the Ag nanoclusters stabilized by POMs (e.g., **Ag27**, **Ag30′**, and **Au8Ag26**) remained essentially unchanged. This indicates that their structures and electronic states are well maintained in organic media.^[^ ^33^, ^36^, ^43^ ^]^

ESI‐mass spectrometry is another useful technique for examining the structural integrity of nanoclusters in solutions. For example, Li and Zheng showed that the ESI‐mass spectra of {Ag_8_} nanocluster stabilized by polyoxoniobates displayed several sets of signals assignable to the nanocluster across a wide pH range (pH 1.3–13.3), supporting its structural stability in aqueous solution.^[^ ^47^ ^]^

XAFS analysis is one of the most powerful techniques for assessing stability and structural/electronic changes of metal nanoclusters, as it can be applied to various sample states (e.g., solid, solution, or supported materials) and provides element‐specific insights into both structures and electronic structures. For example, in situ K‐edge XAFS studies on **Ag30′** during catalytic hydrogenation reaction of nitroarenes revealed that the {Ag_30_} nanocluster of **Ag30′** retained its FCC‐type structure within the ring‐shaped POM framework even under catalytic conditions.^[^ ^36^ ^]^

## 4 Solid‐State Synthesis of Metal Nanoclusters Using POM‐Defined Scaffolds

### 4.1 Ag Nanoclusters within POM‐based Porous Ionic Crystals

Ionic crystals formed by POMs and macrocations often exhibit porous structures. The dimensions, shapes, and sizes of these structures can be finely tuned by selecting specific POMs, countercations, reaction conditions, and additives.^[^ ^51^ ^]^ These porous ionic crystals are typically negatively charged and contain exchangeable cations (i.e., K^+^, Cs^+^, NH_4_^+^), making them promising for applications in proton‐conductive materials and cation‐exchange systems. Notably, unlike other porous crystalline materials such as zeolites and metal–organic frameworks, porous ionic crystals exhibit distinctive redox activity, characterized by the incorporation and removal of cations accompanied by reversible and multistep redox processes of POMs.

**Figure 8:**
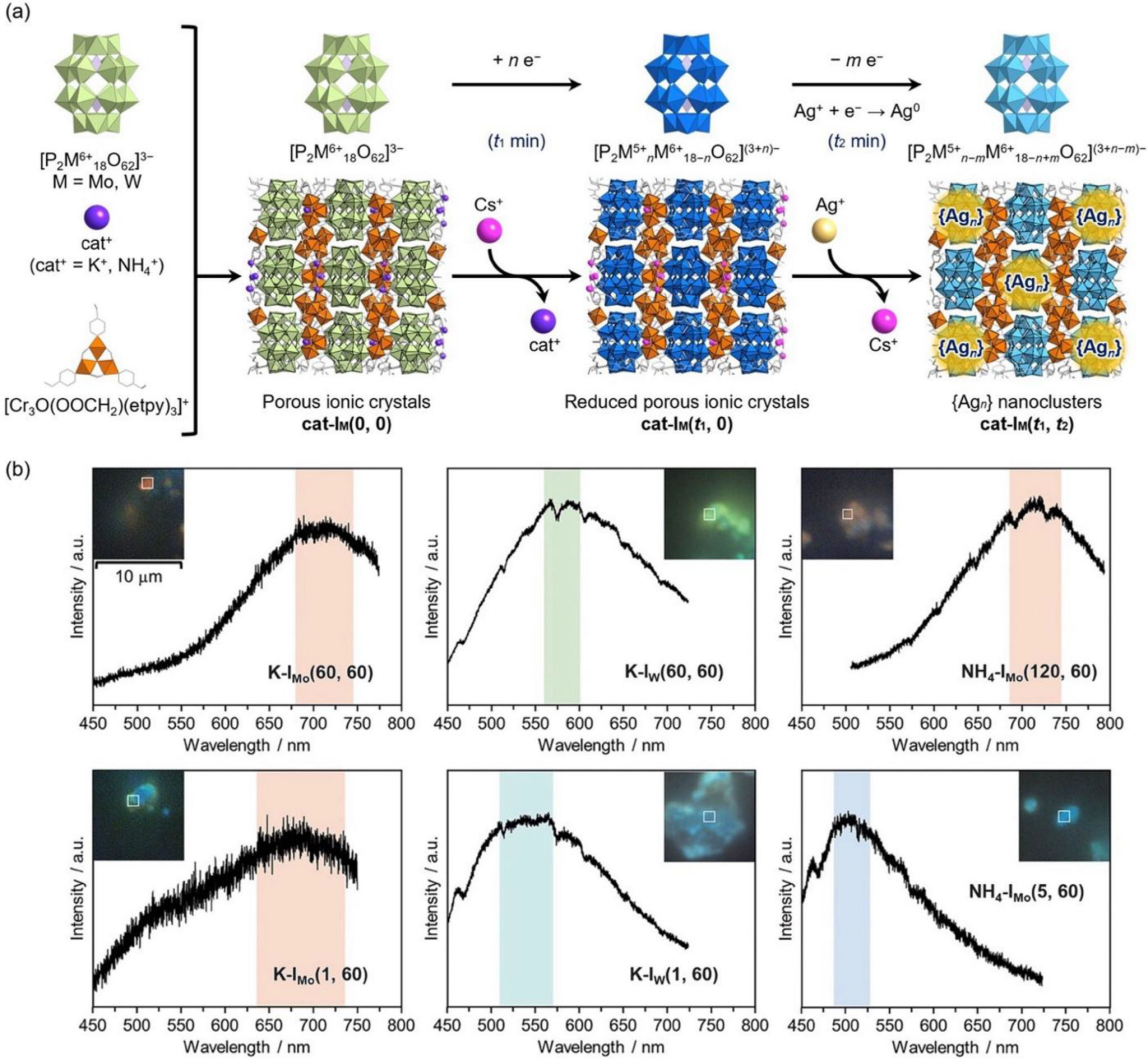
(a) Schematic representation of the synthesis of small {Ag*_n_*} nanoclusters within the scaffold of the porous ionic crystal of [P_2_M_18_O_62_]^3−^ anions (**cat‐I_M_(*t*_1_, *t*_2_)** represents the porous ionic crystal comprising Dawson‐type POM [P_2_M_18_O_62_]^3−^ (M = Mo, W), countercations (cat; cat^+^ = K^+^, NH_4_^+^), and the macrocation, which has been reduced and ion‐exchanged with Cs^+^ for *t*_1_ min followed by the reaction with Ag^+^ for *t*_2_ min). Color code: green octahedra, {MO_6_}; purple tetrahedra, {PO_4_}; orange octahedra, {CrO_6_}; purple sphere, countercations; pink sphere, Cs, light yellow sphere, Ag. (b) Photoluminescence images and spectra of {Ag*_n_*} nanoclusters. Reproduced from the cited literature.^[^ ^52c^ ^]^ Copyright 2023 John Wiley & Sons.

Thus, these porous ionic crystals serve as both well‐defined scaffolds and redox‐active environments that facilitate the synthesis of metal nanoclusters. Recently, Uchida et al. devised a strategy for synthesizing luminescent small {Ag*_n_*} (*n = *3–6) nanoclusters within POM‐based porous ionic crystals (Figure 8a).^[^ ^52^ ^]^ Their two‐step approach involved an initial reduction of Dawson‐type POM [P_2_M_18_O_62_]^3−^ (M = Mo, W) in the as‐synthesized crystals, which simultaneously induced a cation‐exchange process, replacing countercations (potassium or ammonium ions) with cesium ions. In the subsequent step, Ag ions were introduced into the porous ionic crystals via cation exchange, followed by reduction through electron transfer from the pre‐reduced POMs. Spectroscopic analyses, including photoluminescence spectroscopy, X‐ray photoelectron spectroscopy, and XAFS analysis, confirmed the formation of small {Ag*_n_*} nanoclusters; the average number of Ag atoms (*n*) was controllable depending on the choice of POMs, countercations, and reaction periods in each steps, enabling the control of their photoluminescence properties (Figure 8b).

### 4.2 Cu Nanoclusters within Ring‐Shaped POMs

**Figure 9:**
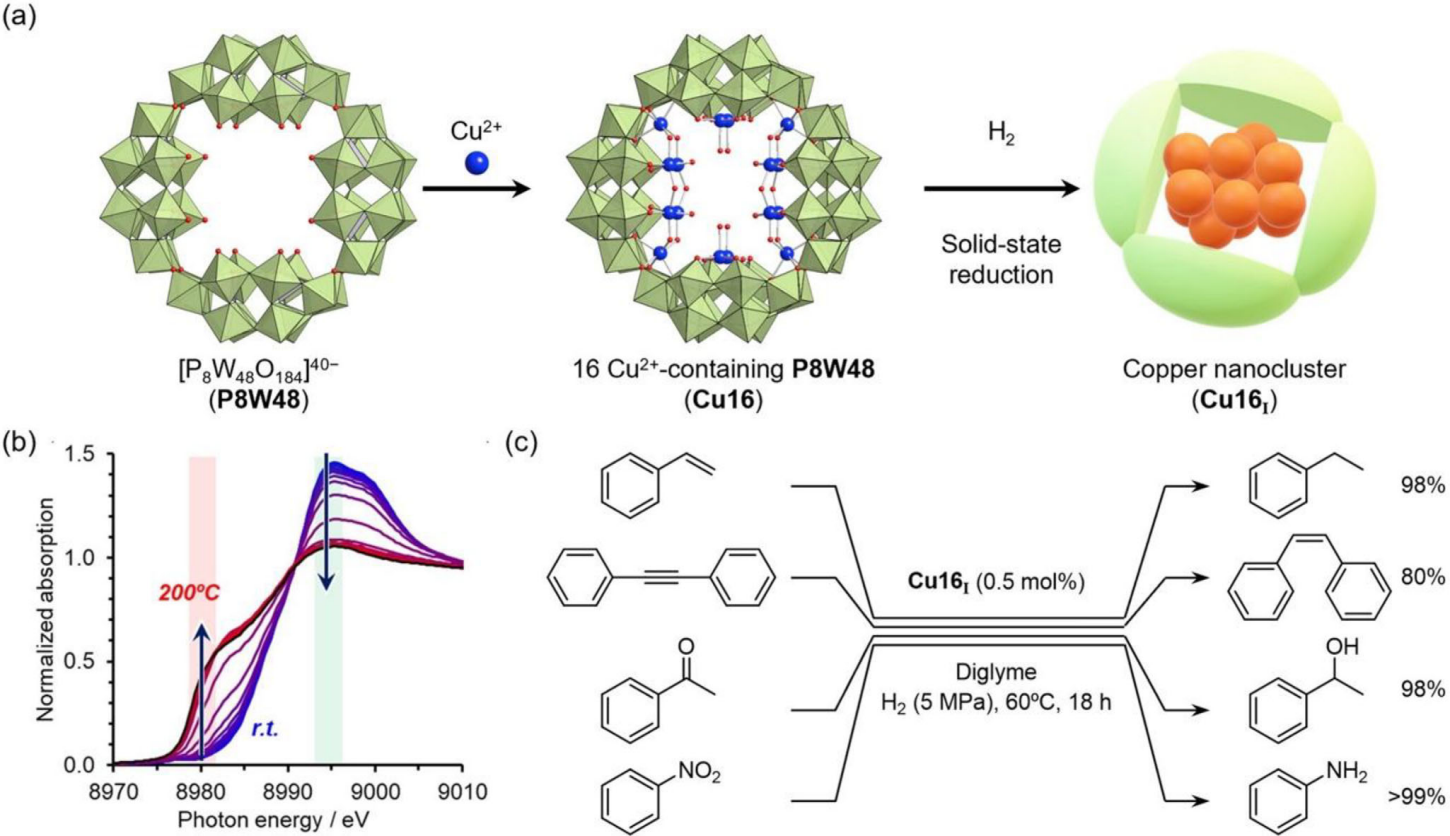
(a) Schematic illustration of Cu nanocluster formation within **P8W48** via the hydrogen reduction of **Cu16** in the solid state. The figure of **Cu16** was made based on the coordinates obtained from the cited literature.^[^ ^53^ ^]^ Color code: green octahedra, {WO_6_}; purple tetrahedra, {PO_4_}; blue sphere, Cu; red sphere, O. (b) In situ Cu K‐edge XANES spectra recorded during the hydrogen reduction of **Cu16** at elevated temperatures up to 200 °C under an H₂ flow. Reprinted from the cited literature.^[^ ^54^ ^]^ Copyright 2024 American Chemical Society. (c) Catalytic hydrogenation reactions by **Cu16_I_**. Reaction conditions: **Cu16_I_** (0.5 mol%), substrate (0.1 mmol), diglyme (2 mL), H_2_ (5 MPa), 60 °C, 18 hours.

The ring‐shaped **P8W48** anion also provides an intrinsic nanosized cavity suitable for hosting metal species. In a recent study, we demonstrated the solid‐state synthesis of Cu nanoclusters with controlled nuclearity via the hydrogen reduction reaction of Cu^2+^‐incorporated **P8W48**. By reacting **P8W48** and Cu^2+^ in organic solvents, we successfully incorporated a defined number of Cu^2+^ cations (*n* = 4, 8, 12, and 16) within the cavity of **P8W48** (**Cu4**, **Cu8**, **Cu12**, and **Cu16**, respectively).^[^ ^53^ ^]^ Crystallographic analysis revealed that these anionic frameworks were spatially isolated by countercations (i.e., TBA) and solvent molecules within the crystal packing. This spatial separation prevented intercluster aggregation and ensured that each Cu oxide unit within the **P8W48** molecules remained structurally distinct. Consequently, the **P8W48** framework acted as a confined reaction vessel, enabling precise control over the size and nuclearity of Cu nanoclusters (**Cu16_I_**; Figure 9a). In situ Cu K‐edge X‐ray absorption near‐edge structure analysis revealed that Cu^2+^ in **Cu16** underwent reduction at temperatures above 140 °C under H_2_ flow (Figure 9b), a considerably lower temperature than the reduction temperature of bulk CuO.^[^ ^54^ ^]^ Curve‐fitting analysis of the extended XAFS of **Cu16** after the reduction process revealed a Cu∙∙∙Cu coordination number of 2.4 ± 0.2, indicating the formation of small Cu nanoclusters (i.e., **Cu16_I_**). In contrast, **Cu4** was barely reduced by hydrogen even at 200 °C.

The Cu nanocluster derived from **Cu16** exhibited catalytic versatility in hydrogenation reactions, demonstrating activity toward substrates such as styrene, diphenylacetylene, benzaldehyde, and nitrobenzene (Figure 9c).^[^ ^54^ ^]^ Importantly, the Cu nanoclusters synthesized within the ring‐shaped POM (i.e., **Cu16_I_**) achieved the catalytic hydrogenation of alkynes while retaining their size within the ring‐shaped POM. The previous reports, where Au nanoclusters have been used as catalysts for semi‐hydrogenation of alkynes, have revealed that this reaction was strongly prohibited by stabilizing ligands coordinating to the Au surface (i.e., thiolates).^[^ ^55^ ^]^ Considering that the removal of organic ligands (e.g., thermal treatment) potentially causes undesirable structural changes and agglomerations of nanocluster catalysts, this result demonstrated that POMs could act as molecular templates for metal nanoclusters imparting unique catalytic activity and enhanced structural stability.

## 5 Summary and Outlook

In POM‒metal nanocluster hybrids, POMs serve as versatile structural and functional components by acting as anionic templates, stabilizing ligands, and porous materials. This versatility stems from the unique structural adaptability of POMs, which can be precisely tailored by modifying their constituent atoms, spatial arrangements, and electronic states. Recent advancements have underscored the pivotal role of basic POM derivatives—such as lacunary POMs and polyoxoniobates—in directing the formation of atomically precise metal nanoclusters. Additionally, oligomeric lacunary POMs exhibit remarkable structural tunability and sufficient flexibility, enabling size‐controlled nanocluster formation and facilitating structural transformations.

Hybridization of metal nanoclusters with POMs has been reported to impart distinctive properties and functionalities, including the following:

1. Enhanced stability: Despite the generally weak interaction between noble metal atoms (soft acids) and oxygen‐rich POM frameworks, POM coordination often provides exceptional stability to metal nanoclusters. In particular, ring‐shaped POMs, such as **P8W48**, offer steric protection while maintaining a partially exposed metal surface.
2. Electronic modulation: The electronic states of metal nanoclusters are strongly influenced by electron donation from anionic POM ligands. This electron transfer can be further modulated by the protonation state of the POMs, leading to pronounced changes in photochemical and optical properties.
3. Synergistic reactivity: The POM‒metal nanocluster hybrids integrate the reactivity of metal nanoclusters with the intrinsic physicochemical properties of POMs, such as acidity/basicity, redox activity, and photochemical behavior. For instance, Ag nanoclusters stabilized by polyoxotungstates exhibit cooperative H_2_ dissociation, where protons and electrons are separately stored within the POMs and Ag nanoclusters, respectively. This unique property facilitates their high selectivity in hydrogenation catalysis. Additionally, POM‐stabilized Ag nanoclusters have demonstrated promising photocatalytic and electrocatalytic performance.

To date, a wide variety of atomically precise metal nanoclusters, including Au, Ag, Pt, Pd, Cu, and alloy nanoclusters,^[^ ^11^, ^56^ ^]^ have been developed using organic ligands as stabilizers. While the synthesis of POM‐stabilized nanoclusters has primarily focused on Ag systems, the development of broader synthetic strategies to accommodate a wider range of metal or alloy nanoclusters remains a significant challenge. This includes improving the compatibility of POMs with metals that exhibit weaker affinities for oxygen donors and achieving precise control over nuclearity, atomic arrangement, and electronic states. As discussed above, for example, ring‐shaped POMs (e.g., [P_8_W_48_O_184_]^40−^) can significantly stabilize encapsulated nanoclusters^[^ ^36^, ^39^, ^43^ ^]^ and act as a nano‐sized reaction vessel for the solid‐state synthesis of Cu nanoclusters,^[^ ^54^ ^]^ highlighting their high potential in both the development and applications of diverse metal nanoclusters. Advanced characterization techniques, such as single‐crystal X‐ray diffraction, high‐resolution mass spectrometry, X‐ray absorption fine structure, and computational studies, will continue to be essential for elucidating structure–property relationships in these hybrids.

In summary, the field of POM–metal nanocluster hybrids stand at an exciting crossroads where molecular‐level design converges with the development of functional materials. Continued interdisciplinary efforts across synthetic chemistry, materials science, and theoretical studies will undoubtedly accelerate the discovery of novel hybrid architectures with tunable properties and wide‐ranging applications in catalysis, energy conversion, sensing, and beyond.

## Conflict of Interest

The authors declare no conflict of interest.

## Data Availability

Data sharing is not applicable to this article as no new data were created or analyzed in this study.
